# Borough of Manhattan, September 5, 6, 7, 1899

**Published:** 1899-05

**Authors:** 


### A. V. M. A.

BOROUGH OF MANHATTAN, SEPTEMBER 5, 6, 7, 1899.

Dr. Charles Gresswell will present a paper entitled "The Veterinarian in Official Life."

Dr. Benjamin McInnes, of Charleston, South Carolina, will contribute some "Notes in Filaria Immitis."

Meat-inspection in all its phases will receive the most extensive consideration at New York, and the display of pathological specimens found in the slaughter-houses will exceed that of any similar exhibition at any of our meetings. Those who had the pleasure of visiting Omaha will recall with great pleasure the unusual demonstration there.

Dr. S. J. J. Harger, of Philadelphia, has consented to operate in the clinic at the coming meeting, and will, among other operations, demonstrate the different methods advocated to-day for correction of the cribbing habit.

Resident State Secretary Ridge, of Pennsylvania, is fully alive to the work of having the Keystone State well represented at New York. He starts out with an unusual delegation from the State Association.

Every city in the land should send a representative veterinarian to participate in the discussion and consideration of the subject of meat-inspection. Every Board of Health should send a duly accredited delegate to become more enlightened upon what is possible to attain and what success has followed this work at home and abroad.

Several Tennessee veterinarians have already notified Resident Secretary Plaskett of their intention to attend the annual meeting in New York City.

The following appointments of Resident State Secretaries have been made by the President, Dr. A. W. Clement: Colorado, Dr. Charles Gresswell, Montclair, Denver; Wisconsin, Dr. R. H. Harrison, 206 Thirteenth Street, Milwaukee; Kansas, Dr. W. H. Richards, Emporia; Maryland, Dr. E. C. Fox, 2823 Huntingdon Street, Baltimore; Massachusetts, Dr. Benjamin D. Pierce, 27 Sanford Street, Springfield. The last three appointments were to fill vacancies caused by resignation.

Resident Secretaries are actively at work in the interests of the Association and the coming meeting. Through their efforts a number of papers have been promised for the programme. Dr John J. Repp, of Ames, Iowa, and Dr. W. Horace Hoskins have signified their intention to contribute papers. Dr. C. C. McLean, of Meadville, will present a paper on "Dairying from a Pure Milk Stand-point," with a practical demonstration of milk-testing.

Among the features of the 1899 meeting will be extensive surgical clinics to be participated in by Drs. Gill, of New York; Adams and Harger, of Philadelphia, and a number of others whose names are not yet ready to be announced. There will be several operating-tables exhibited, and this plan of securing animals will be fully demonstrated. The various methods of casting and confining animals will be demonstrated by those who have given this special attention. The demonstration of anæsthetics will be practised, and any members of the profession who desire to take part in any of this work will kindly notify Dr. H. D. Gill, chairman of the Committee of Arrangements, 154 East Fifty-seventh Street, New York City. Veterinary societies adjacent to New York—State and local—will all be asked to send a number of delegates to participate in the coming convention. Pennsylvania has already planned to send ten delegates to represent their State Association. A number of side-trips in and around New York City will afford visiting members much pleasure and an opportunity of seeing much that Greater New York has to show.

Dr. William H. Lowe, Treasurer of the Association, has consented to further participate in the programme at New York in September, and will present a paper entitled "Routine Manipulations and Operations"—a subject of every-day importance, and bound to be eminently worthy of a place on the programme, coming from one who has attained a successful place among the body of general practitioners.

President A. W. Clement has appointed Dr. H. D. Gill chairman of the Entertainment Committee for the coming meeting of the Association in New York City. As associates he will have the valued assistance of Drs. George H. Berns, R. R. Bell, E. B. Ackerman, and W. H. Pendry.

Several other association meetings will be held in conjunction with the 1899 meeting. Among others, the New York State and New York County Veterinary Medical Associations.

In conjunction with the gathering in New York in September there will be held the twenty-fifth anniversary of the American Veterinary College. President Pendry, of the Alumni Association, is planning for a grand reunion of the A. V. C. boys.

